# Cognitive arousal-based measures quantify insights from self-ratings in response to sensory stimuli

**DOI:** 10.1371/journal.pmen.0000463

**Published:** 2025-11-12

**Authors:** Suzanne Oliver, Jinhan Zhang, Vidya Raju, James W. Murrough, Rose T. Faghih

**Affiliations:** 1 Department of Mechanical and Aerospace Engineering, New York University (NYU) Tandon School of Engineering, Brooklyn, New York, United States of America; 2 Department of Electrical and Computer Engineering, New York University Tandon School of Engineering, Brooklyn, New York, United States of America; 3 Department of Biomedical Engineering, New York University Tandon School of Engineering, Brooklyn, New York, United States of America; 4 Department of Psychiatry, Depression and Anxiety Center for Discovery and Treatment, Icahn School of Medicine at Mount Sinai, New York, New York, United States of America; 5 Department of Neuroscience, Icahn School of Medicine at Mount Sinai, New York, New York, United States of America; 6 VISN 2 Mental Illness Research, Education, and Clinical Center (MIRECC), James J. Peters VA Medical Center, Bronx, New York, United States of America; 7 Center for Urban Science and Progress, NYU, Brooklyn, New York, United States of America; PLOS: Public Library of Science, UNITED KINGDOM OF GREAT BRITAIN AND NORTHERN IRELAND

## Abstract

Reliable quantification of patients’ cognitive arousal is a challenging problem and a pertinent clinical need in various mental health applications. Recently, skin conductance-based cognitive state estimation has shown promise in inferring the cognitive arousal of individuals caused by autonomic nervous system (ANS) activation. Here, we use a physiological model of ANS-stimulated skin conductance modulations and Bayesian filtering to analyze changes in cognitive arousal induced by auditory, visual, and haptic stimuli. Our findings indicate that cognitive arousal-based measures are in better agreement with self-ratings-based metrics than inferred autonomic nervous system activation events in response to sensory stimuli. These insights on cognitive arousal increase our understanding of psychophysiology and may help diagnose, track, and treat symptoms of mental health disorders in the future by providing clinicians with a framework to estimate and modulate arousal levels in an interactive sensory stimulation environment.

## Author summary

In this work, we find that measures derived from continuous skin conductance measurements effectively convey insights on self-perceived arousal in response to visual, auditory and haptic stimuli. The conclusions from this analysis will contribute towards the quantification, tracking and eventually modulation of cognitive states during human-computer interactions (e.g., workplace or virtual reality settings) for entertainment as well as for non-invasive, non-pharmacological closed-loop mental health interventions.

## Introduction

Human emotions can be characterized by independent components such as cognitive arousal (the intensity of autonomic nervous system activation due to cognitive processes invoked in emotion perception such as memory, attention, sensory processing, etc.), valence (the positive or negative characteristics of the emotion), and dominance (the level of control felt during the emotion) [1,2]. Higher intensity of autonomic nervous system (ANS) activation and, therefore, higher cognitive arousal can be a result of exposure to fearful or stressful stimuli, pain, or as a symptom of mental health conditions such as post-traumatic stress disorder or anxiety disorders [3,4]. In patients suffering from cognitive symptoms, such as brain fog from post-acute sequelae of COVID, quantification of the arousal may help indicate alterations in cognitive functioning due to disease or age-related cognitive decline [5]. Activation of the ANS and its modulation through interventions including music, breathing exercises, or other sensory stimuli can have a substantial impact on people, affecting their emotions, cognition, and ability to focus [2,6–9]. This can offer non-pharmacological ways to improve productivity in the workplace or academic environments and, more broadly, mental health.

Recent works have attempted to regulate emotional or cognitive states of participants using various non-pharmacological interventions. In [9], the temporal cognitive arousal and performance variations during working memory tasks were quantified in response to music (neutral, relaxing, or exciting), perfume, and coffee. This work also shed light on the participant-specific optimal range of cognitive arousal for which performance is maximized according to the Yerkes-Dodson law, with applications in improving workplace productivity [10,11]. Multi-sensory stimulation has been shown to alleviate symptoms of cognitive decline in individuals with dementia [12]. For patients suffering from chronic pain with unpredictable flare-ups, immersive sensory experiences are recently emerging as a potent intervention to offer a reprieve from the ever-present pain and associated mental health consequences [13]. Additionally, pain in infants in intensive care units has been shown to be reduced with exposure to familiar sounds and smells [14], and music therapy has the potential to be an effective pain management tool in surgical patients [15]. Stimulating sensory environments that invoke a sense of awe have been shown to have positive physical and mental health benefits [16–19].

Thus, there is a clear need to quantify changes in the cognitive states through the measurement of peripheral physiological signals, as the ability to monitor and modulate these states could allow for improved closed loop design of non-invasive interventions.

Virtual reality (VR) platforms have emerged as a viable intervention to regulate emotions and cognition. For example, VR has been shown to have the ability to induce various types of emotions in participants in laboratory settings [20–22]. VR has also been shown to be effective in mitigating severe pain in hospitalized patients [13,19].

The combination of sensory stimuli available in VR can create a desired immersive environment for the user, effectively modulating their cognitive state. For this reason, it is critical to develop an understanding of how different stimuli affect VR users to ensure that the desired cognitive state modulation is achieved. Thus, there is a need for non-invasive sensing and computational methods for determining the arousal level of an individual and exploring the effectiveness of each type of sensory stimulus in eliciting cognitive arousal. Understanding how arousal levels change in individuals with various afflictions in response to different sensory input modalities can help improve the non-pharmacological interventions.

One candidate modality for non-invasive estimation of arousal is skin conductance (SC), which is reflective of the sweat secretion activity and can be measured through sensors on the surface of the skin. Fast, small amplitude changes in SC can provide information pertaining to ANS activation in response to psychological stress, cognitive loads, or environmental stimuli. Many signal-processing methods have been used in previous work to analyze the SC measurements. One early method was a heuristic sigmoid-exponential method to characterize the SC response [23]. However, this type of model cannot recover the activations underlying the rises in the SC signal. Differential equation methods, such as in [24] use bi-exponential functions as solutions to capture only the fast-varying phasic component of SC measurements. Other work separates this phasic component and the slow-varying tonic component with Finite Impulse Response (FIR)-based filters before processing them [25]. These methods aim to separate tonic and phasic components and process each accordingly. However, this separation can be better achieved with deconvolution methods. Deconvolution has been used to separate phasic and tonic components and recover the parameters for different mathematical models, but there was a lack of consideration for the actual physiology of the ANS response that triggers SC peaks [26]. To address these limitations, a physiologically plausible method for inferring the ANS activation from SC has recently been proposed in [27–29]. In this approach, the ANS system is modeled as the input to a state-space model with the SC values as the measurable output. The model embeds the physiologically valid assumption that ANS pulses result in secretions of sweat in the sweat glands. These secretions increase the pressure in the gland and can either slightly increase the SC through diffusion or, if the pressure in the gland exceeds the threshold, cause the pore to open and sharply increase the SC. Expectation-maximization is used to decode the activation pulses of the ANS system. This model allows for the separation of the phasic and tonic components of the SC response and places constraints on the model parameters to ensure they are biologically plausible. Other options for this skin conductance analysis include SCRalyze, Ledalab, and CVXEDA [30,31]. Ledalab and CVXEDA were directly compared with this method in [27] and found to be less accurate at distinguishing between high arousal and low arousal stimuli.

In this work, we quantified the effect of different types of sensory stimuli in an interactive VR setup on the ANS response and cognitive arousal of healthy adults by analyzing skin conductance data collected from 100 participants in [32]. Specifically, we hypothesized that the change in cognitive arousal will be affected by the type of stimulus and the participant’s experiences. The aims of this study were to: (1) estimate the ANS activation in response to the stimuli and the parameters of a model that describes its effect on the physiology, using the method described in [27], (2) investigate the timescale of ANS activation response to stimuli, (3) compare the quantified ANS activation responses for different stimulus types, (4) estimate the underlying cognitive arousal state in response to each stimulus, (5) validate changes in cognitive arousal estimate against self-reported measures, and (6) identify the sensory modality that caused the highest level of cognitive arousal.

## Materials and methods

### Ethics statement

We used data from [32] (accessed on August 31, 2023 for research purposes), where 100 healthy participants (age range: 18–71 years, mean*±*SD = 26.88 *± *9.11 years) participated in the study, which was approved by the University of Sussex ethics committee and adhered to the principles of the Declaration of Helsinki. The authors did not have access to any identifying information of individual participants during or after the data collection from [32]. All participants provided written informed consent and were financially compensated for their participation.

### Experimental setup and dataset description

**Fig 1:**
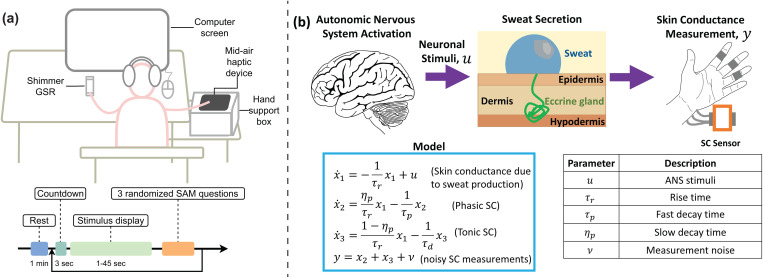
Overview of experimental setup and physiological modeling approach. (a) Experiment setup showing (top) a participant ready to receive an auditory, visual, or haptic stimulus while their skin conductance (SC) is recorded and (bottom) the timeline of the experiment. (b) Physiological model of SC variations caused by ANS activation. Here, the ANS activity (i.e., cumulative neuronal pulsatile signaling to sweat glands) is modeled as the input to a state-space model with the SC values as the measurable output, and the sweat secretion parameters and ANS activity are estimated using an iterative expectation-maximization approach. The recovered ANS activity is then used to estimate the cognitive arousal using a statistical model. The model is described in detail in S1 Text.

In this work, we used skin conductance data from human participants who were exposed to different stimuli, as described in [32]. Of the total 100 participants, 61 were female and 39 were male, and 9 participants were left-handed while the other 91 were right-handed. All of them had normal vision with or without the help of vision correction and none of the participants had any auditory or tactile disorders. The participants did not have any psychological, psychiatric or neurological disorders. The participants were exposed to three types of stimuli: visual, auditory, and haptic, in a randomized order while their SC was continuously recorded. A visual representation of the experimental setup is provided in Fig 1. Skin conductance measurements were taken on the fingers of the left hand using a Shimmer3 GSR+ Unit (Shimmer Sensing, Dublin, Ireland) at a rate of 512 Hz. Before starting the experiment, participants were instructed to rest for 60 seconds. Of the 100 total participants, 24 were rejected due to noisy or incomplete data (see Table A in S1 Table for details). Of the remaining 76 participants, 31 were male and 45 were female (age range: 18–71 years, mean*±*SD = 27.16 *± *9.43 years).

Each participant was exposed to fifty different stimuli, of which twenty were auditory, twenty were visual, and ten were haptic. The durations of the exposure to each stimulus varied, with median lengths of 9.5 *± *14.6, 15.0 *± *0.0, and 1.3 *± *0.3 seconds, respectively, for the audio, visual, and haptic stimuli. All visual stimuli were shown for fifteen seconds each. For the auditory stimuli, the length of exposure was dependent on the particular stimulus to allow rhythms and melodies to conclude. All haptic stimuli were completed under three seconds. The onset time for each stimulus was recorded. The stimuli were chosen to cover a range of expected valence and arousal ratings. After each stimulus, participants rated their emotional state using the Self-Assessment Manikin (SAM) scale [33]. This scale uses pictorial representations of arousal, valence, and dominance and is considered the best representation of participants’ emotional affect states. The participants were exposed to a total of twenty audio stimuli, ten of which were from the International Affective Digitized Sounds (IADS), and the remaining were instrumental music. Similarly, out of the twenty total visual stimuli, ten were chosen from the International Affective Pictures System (IAPS), and the remaining ten were images of abstract visual art. There were ten total haptic stimuli previously determined to evoke emotional responses.

Visual stimuli were shown on a screen placed directly in front of the participant at a distance of forty centimeters. The auditory stimuli were given through a pair of Beats headphones (Beats Studio, Culver City, CA, USA) at a maximum volume of ninety decibels. The haptic stimuli were provided using a mid-air haptic system developed by Ultrahaptics Ltd (Mountain View, CA, USA). The device contained a 4 *× *4 grid of ultrasonic transducers, which could be activated individually or simultaneously at three levels of intensity and frequencies ranging from sixteen to 256 Hertz. Although nine of the participants were left-handed, all participants received the haptic feedback to their right hand.

After each stimulus, the users were asked to self-report their valence, arousal, and dominance using the SAM metric on a scale of 0–100. The order of these three questions was randomized for each stimulus, and no time limit was given to answer the questions. In this study, we only considered the responses to the arousal question. After the SAM questions were completed, a three-second countdown began before the start of the next stimulus.

### Data pre-processing

The dataset consisted of preprocessed skin conductance data: the raw skin conductance data was preprocessed using the MATLAB Ledalab toolbox, as outlined in [32]. With this toolbox, the data was visually inspected for discontinuities from motion artifacts, which were corrected using spline fitting. For this paper, we low-pass filtered this preprocessed data using a minimum-order filter with a stopband attenuation of 60 dB and a cutoff frequency of 2 Hz. The data was then downsampled to 4 Hz to reduce computational cost during processing.

Some participants were rejected due to excessive noise, large periods of discontinuity in the skin conductance data, or incomplete data. A full list of the rejected participants and the reason for removal can be found in the Table A in S1 Table. In total, fifteen participants were rejected due to poor data quality (high noise or regions of disconnection), and nine were rejected due to incomplete recordings, where the skin conductance recording length was much shorter than the time period over which the stimuli were applied. Thus, we proceeded with the analysis for the remaining seventy-six participants with complete, good-quality data.

### Approach

**Fig 2:**
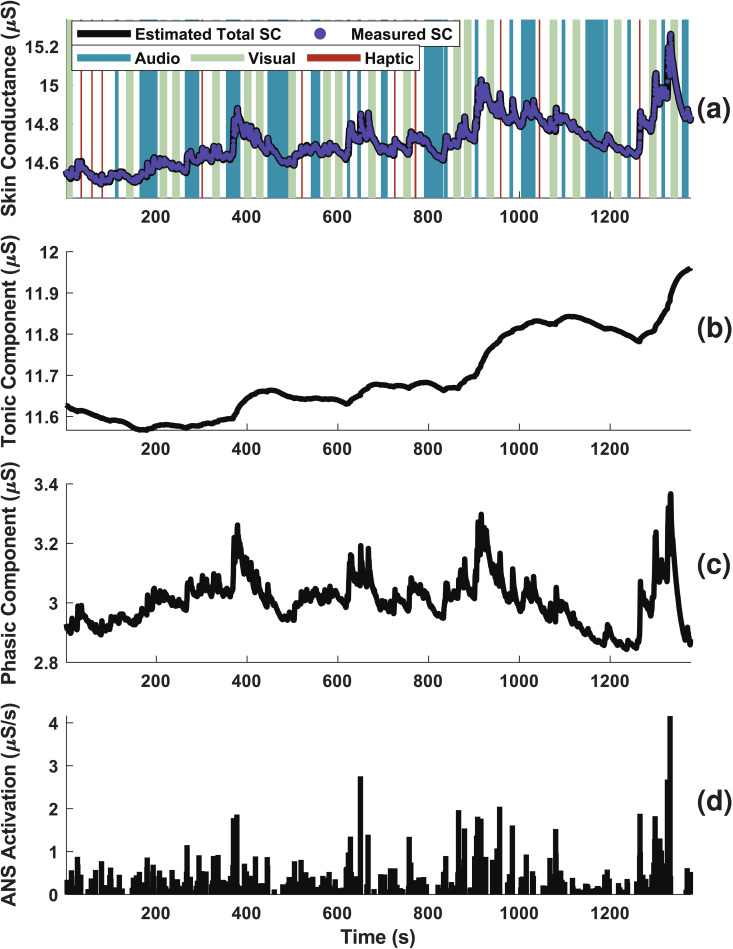
Deconvolution results for participant 1. (a) Skin conductance measurements and estimate, (b) tonic component, (c) phasic component, and (d) ANS response for participant 1 throughout the experiment. In (a), the stimulation events are also shown, with auditory stimuli in teal, visual stimuli in light green, and haptic stimuli in maroon.

To uncover the ANS response, we used the state-space and deconvolution approach outlined in [27] and summarized in Fig 1 to recover the individual-specific ANS response. Using the recovered sweat secretory parameters and input (ANS response) of the model, we reconstructed the timing and magnitude of the sparse ANS responses throughout the experiment. An example of this process is shown in Fig 2 for the participant 1 results. The figure shows the (a) skin conductance values, the decomposition into (b) tonic and (c) phasic components, and the (d) estimated ANS response pulses.

**Fig 3:**
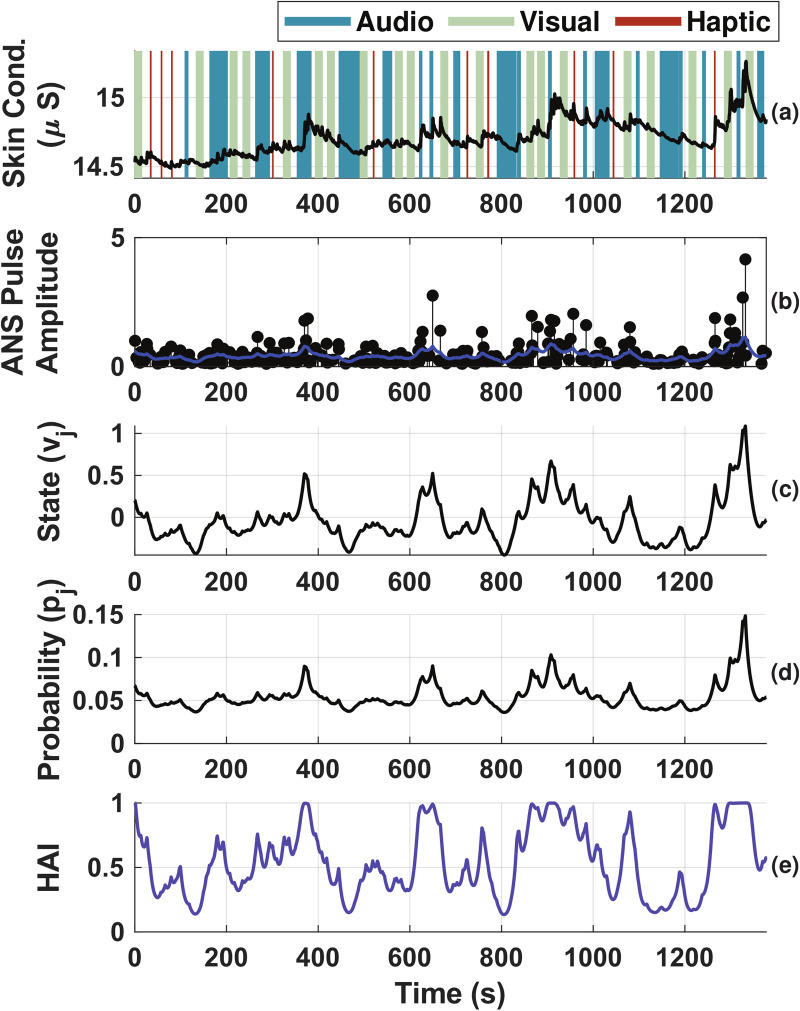
Cognitive arousal estimation results for participant 1. (a) Timing of stimulus, color-coded by type and measured skin conductance measurement, (b) ANS response timings, (c) estimated arousal state, (d) probability of pulse, and (e) High Arousal Index for Participant 1.

The pulses were then used as the input to a Marked Point Process-based algorithm. This method, recently developed in [1], computed a continuous arousal state estimate for each user using the timing and magnitude of the ANS response pulses. The results for participant 1 are shown in Fig 3. Fig 3(c) shows the arousal estimate from the ANS pulses shown in panel (b). Panel (d) shows the probability of an ANS pulse at each time step, and panel (e) shows the High Arousal Index (HAI), which is the probability that the participant’s arousal state is above their median level during the data collection period. The probability and HAI can both be mapped from the arousal state estimate, and are included here to show different ways to visualize the arousal information. An expectation-maximization approach was again used to estimate the arousal and compute participant-specific model parameters. Statistical comparisons of inferred parameters were done using the Wilcoxon signed-rank test. These methods are elaborated in S1 Text.

### ANS stimuli analysis

#### Norms.

To evaluate how the ANS response changed during different periods of the experiment, we considered three norms of the ANS pulses. Let $\begin{array}{c} \vec{\text{u}}=\;[{\text{u}}_{1}\;{\text{u}}_{2}...\;{\text{u}}_{n}{]}^{\prime } \end{array}$ represent a vector in R^*n*^ whose elements u_*i*_, i = 1,..., n represent the amplitudes of the pulses at each sampling time for a total of n samples. The 0-norm is the number of pulses in the total observation time period ($\begin{array}{c} {\left\|\vec{\text{u}}\right\|}_{\text{0}}=\text{card}\left\{{\text{u}}_{i}|{\text{u}}_{i}\neq \text{0}\right\} \end{array}$, where card is set cardinality), the 1-norm is the sum of all pulse amplitudes (note that all pulses are non-negative) in the period (${\left\|\vec{\text{u}}\right\|}_{\text{1}}=\Sigma \left|{\vec{\text{u}}}_{i}\right|$), and the 2-norm is the square root of the sum of the squared value of each pulse amplitude ($\begin{array}{c} {\left\|\vec{\text{u}}\right\|}_{\text{2}}=\sqrt{\Sigma_{i}u_{i}^{\text{2}}} \end{array}$) Each of these norms was computed during four different sections of the experiment. These three norms were chosen because they each give important information about the ANS pulse events. The 0-norm indicates the number of pulses, the 1-norm indicates the sum of amplitudes of the pulses, and the 2-norm is the sum of the squares of the pulse amplitudes, or ‘energy’, which is more affected by larger pulses than smaller ones.

These three metrics together encode important information about the ANS response.

First, norms for the periods within two seconds of a new stimulus onset and, separately, the periods within five seconds of a new stimulus onset were computed. (Note that the full two- or five-second period after the onset of the stimuli was considered even for stimuli lasting less than five seconds). Next, the norms were computed for any time when a stimulus was activated. Finally, to consider the effect of the stimuli compared to the absence of stimuli, the norm was computed for the periods when no stimuli were active. This computation also excluded the three-second period leading up to a new stimulus since participants were shown a visual countdown to the next stimulus at this point, which could impact the results. Moreover, the two-second after stimuli onset region was further broken down by stimulus type. So, the norms for the two-second periods following the onsets of visual, haptic, and auditory stimuli were considered separately. The distributions of these norms across participants were found to be non-normal (per the Kolmogorov-Smirnov test), so the Wilcoxon signed-rank test was used to evaluate statistically significant changes between them.

#### Transitions in arousal.

The stimuli were grouped into three sections based on their mean SAM scores across participants. Stimuli above the high cutoff percentile for SAM scores were considered High-Rated stimuli, stimuli between the low cutoff and high cutoff percentiles were Mid-Rated stimuli, and below the low cutoff were Low-Rated stimuli. To investigate whether this model can capture changes in arousal level, the transition points (ex., when a participant goes from a Low-Rated to a Mid-Rated stimuli) between these groups were considered.

At each transition point, arousal state estimates were considered for the stimuli immediately before and after the transition. Specifically, the mean arousal estimated for the first two seconds after stimulus onset was computed. An increase in arousal estimate is expected when transitioning from a lower-rated stimulus to a higher-rated stimulus. Thus, on these transitions (going from Low-Rated to Mid- or High-Rated, or Mid-Rated to High-Rated), if the arousal estimate increased from the first to the second stimuli, this was considered a success. Similarly, for the transitions from higher-rated to lower-rated (High-Rated to Mid- or Low-Rated or Mid-Rated to Low-Rated), a decrease in arousal estimate was a success. Every successfully predicted transition between each type was summed (across all participants) and divided by the total number of transitions of that type to compute the accuracy of capturing these transitions.

To evaluate the robustness of this method, various values were tested for the high and low transition cutoffs, ranging between the 55^*th*^ and 95^*th*^ percentiles for the high cutoff and 5^*th*^ and 45^*th*^ percentiles for the low cutoff at 5% increments.

#### Identifying high arousal stimuli.

To consider which type of stimuli had the largest impact on the arousal of the participant, we isolated the stimuli that coincided with the highest levels of HAI. To do so, the fifteen stimuli that occurred during the highest level of HAI for each participant were identified and grouped by stimulus type: auditory, visual, or haptic. This group was named the ‘high HAI set’. There would be a bias against the haptic stimuli in this method because those stimuli were only one to two seconds in length since (if the stimuli types all had the same effectiveness) they would be less likely to coincide with the high arousal regions than the longer-lasting stimuli. To account for this bias, for any stimulus lasting less than five seconds, the five-second region after the stimulus onset was considered part of the stimulus exposure time. A duration of five seconds was chosen to match the shortest of the auditory stimuli while still limiting the period to immediately after the stimuli exposure.

For each participant, the number of stimuli of each type in the high HAI set was summed and divided by the total number of stimuli of that type (twenty for auditory and visual; ten for haptic). These ratios for all participants were then compared to consider which stimuli type most often resulted in high HAI. The composition of the high HAI set was also compared to the self-reported SAM ratings. The participants were grouped into two sections: those that had a higher mean SAM arousal score for haptic stimuli compared to visual, and those that did not. The proportion of each type in the high HAI set was compared separately for these two groups. A Kolmogorov-Smirnov test was done on the distributions of ratios, which rejected the null hypothesis that the data is normally distributed. As a result, Wilcoxon signed-rank tests were performed to compare the statistical significance of differences in the distributions.

#### Comparison to SAM values.

The self-reported SAM arousal values were considered for each participant. The participants were asked to rate on a scale of 0–100, but it was noted that many participants used only a subset of this range. To eliminate this difference and make it easier to compare between participants, the SAM values were normalized for each participant using linear scaling to fill the 0–100 range. In addition to comparing the stimuli in the high HAI set by type, we compared the individual stimuli to their SAM values. In this analysis, the SAM values were considered as the ground truth, with the stimuli with the highest SAM values being considered high arousal events for that participant.

To assess the accuracy of this method in detecting high arousal events identified by participants, we considered how many of the high arousal event stimuli were also in the high HAI set. Stimuli in both groups were considered true positives, while stimuli identified as high arousal events but that were not in the high HAI set were false negatives. Similarly, stimuli in neither group were true negatives, and stimuli only in the high HAI set were false positives. These values were used to compute the accuracy, sensitivity, and specificity of our method.

The cutoff SAM value to be considered in the high HAI set was determined in a participant-specific manner. This is justified because the self-reported scores are subjective, and the number of true high arousal events may differ between participants. For each participant, we iterated through cutoff values of 50–100 in increments of 1. The cutoff value that resulted in the highest accuracy score (for predicting high arousal stimuli) with at least five stimuli in the high HAI set was chosen for each participant. The cutoffs ranged from 50 to 99 (median 78 *± *11), resulting in between 5 and 21 (median 7 *± *3) stimuli in the high HAI set. The accuracy results for all participants are presented in S2 Table.

## Results

### Deconvolution

#### Deconvolution explained ANS activation events.

Through the deconvolution approach using the model described in Fig 1, we estimated participant-specific parameters of skin conductance signal (rise time and slow and fast decay times), inferred the ANS activation (times and magnitudes of stimuli inducing sweat secretion), and closely reconstructed the measured skin conductance values. The results for Participant 1 are shown in Fig 2. Fig 2(a) shows both the measured and reconstructed skin conductance values (median R^2^ between these signals for all participants was 1.0000 *± *0.0001). Fig 2(b) and (c) show the component of the skin conductance attributed to the slow-varying tonic and fast-varying phasic components, respectively, the sum of which is the total skin conductance.

The ANS pulses cause the release of sweat, which should immediately increase phasic skin conductance, as can be seen in Fig 2(d). The results for the remaining participants are provided in the S1 Fig–S9 Fig.

#### Highest ANS response occurred within two seconds of stimulus onset.

**Fig 4:**
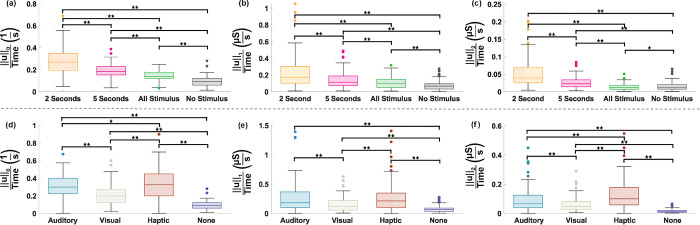
Distributions of norm values of recovered ANS responses, normalized by time. Subplots indicate the (a)(d) 0-norm, (b)(e) 1-norm, and (c)(f) 2-norm. In subplots (a), (b), and (c), the boxes represent different time windows after stimulus onset: two seconds (orange), five seconds (pink), total duration of stimulus exposure (green), and time periods when no stimuli are active (grey). In subplots (d), (e), and (f), the boxes represent different stimulus types: auditory (teal), visual (light green), haptic (maroon), and none (purple). * indicates a significant difference with p < 0.05 and ** indicates a significant difference with p < 0.001 per the Wilcoxon Signed-Rank test.

We analyzed the total number and magnitudes of the recovered ANS pulses during the experiment to consider how the stimuli affected the nervous system by calculating the 0-, 1-, and 2-norms [34]. Fig 4(a), (b), and (c) show the distribution of the total number (0-norm), sum of amplitudes (1-norm), and energy (2-norm) of the ANS pulses for all participants for different segments of the experiment, normalized by the duration of the stimulus exposure in each segment. The time segments considered here were the periods (i) within two seconds of a new stimulus onset, (ii) within five seconds of a new stimulus onset, (iii) time when any stimulus is active, and (iv) time when no stimuli are active. Note that for (i) and (ii), the full two- or five-second period after the start of the stimulus was considered even for stimuli with shorter durations. Significance testing between conditions was done via the Wilcoxon signed-rank test, with a significance level of p < 0.05. These measures were significantly higher for the period of two seconds within a new stimulus onset than for any of the other durations calculated. Additionally, the five-second period had significantly higher ANS pulse norms than the any-stimulus and no-stimuli periods. We found higher 0-norm and 1-norm, but slightly lower 2-norm, for periods when any stimulus was active in comparison with periods of no stimulus.

The Wilcoxon signed-rank test showed significant differences between all pairs of periods for all norms, with p < 0.001 for all tests except for the any-stimulus to no-stimulus comparison of the 2-norm, which had p < 0.05.

To analyze how participant demographics may impact these trends, we ran the analysis separately for males, females, young adults (age < 30), and older adults (age *≥ *30). Statistical testing was done via the Wilcoxon rank-sum test. Comparing the male and female participants, the test failed to reject the null hypotheses (that there is no difference between groups) for any of the norms and time periods tested (p > 0.05). Comparing the results of the two age groups, higher 0-norms were found in the older group than in the younger group for the five-second time period (p < 0.05). The test failed to reject the null hypothesis for all other norms and time period (p > 0.05). It should be noted that the age analysis is limited by the demographics of the participants, with 68% of participants being under 30 years old.

#### Haptic stimuli caused greatest ANS activation.

Since most ANS activation occurred within two seconds of the stimulus onset, we compared the norms for different stimulus types during this period. These results can be found in Fig 4(d), (e), and (f). Again, the Wilcoxon signed-rank test was used to assess statistical significance. There was a significant increase in the norms when any of the stimulus types were applied in comparison to the time periods when no stimuli occurred, as one would expect (p < 0.001 for all stimulus types and norms). The median ANS response was largest for the haptic stimuli. It was significantly higher than for visual stimuli for all norms tested (p < 0.001 for all norms) and significantly higher than the 0-norm (p < 0.05) and 2-norm (p < 0.001) for the auditory stimuli (no difference for the 1-norm (p > 0.05)). The auditory stimuli also produced a greater ANS response than the visual stimuli for all norms tested (p < 0.001 for all norms).

Again, the analysis was run separately for male and female participants, and for older (age *≥ *30) and younger (age < 30) participants, with significance testing done via the Wilcoxon rank-sum test. The female participants had significantly higher 1-norm and 2-norm results for the visual haptic stimuli compared to the male participants (p < 0.05). The older participants had significantly higher 0-norm results for the visual stimuli than the younger participants (p < 0.05). The test failed to reject the null hypothesis for all other comparisons (p > 0.05).

#### Estimation of cognitive arousal: Marked point process-based quantification of ANS activation.

We estimated the cognitive arousal state of the participants from inferred ANS activation pulses by using the Bayesian state estimation framework (see S1 Text and [35]). This model also produces the probability of an ANS pulse occurrence in a given time step and a High Arousal Index (HAI) for the participant, which indicates the probability that the user’s arousal estimate is above their median state estimate. The results for Participant 1 are shown in Fig 3 and for the remaining participants in S10–S18 Figs.

To validate this arousal estimation step, we considered the temporal variations of the estimated state as shown in Fig 3(c) and their relation to the inferred ANS pulses. The state estimate increased when there were high-magnitude ANS pulses or when low-amplitude pulses occurred frequently. Conversely, regions with sparse pulses of low magnitudes corresponded to a decrease in the arousal estimate.

The probability of a pulse occurrence hovered near 0.05, as shown in Fig 3(d). The baseline probability for this participant was 0.056, meaning that when arousal was low, the probability is close to the baseline, as expected. This means we would expect a pulse approximately once in a five-second period, when the user is near their baseline arousal. There were some notable spikes in the probability near 900 and 1300 seconds. At these times, the probability of impulses rose well above the baseline probability.

These periods of high probability aligned with higher arousal in the user and correspond with more frequent ANS pulses. The HAI, shown in Fig 3(e), also varied with the arousal estimate, though it appeared to be more sensitive to small changes in the state estimation than the probability is, effectively capturing both increases in the number and amplitudes of the ANS pulses. For instance, the HAI reached its maximum possible value during several periods of high arousal, such as at 650, 900, 1100, and 1300 seconds. Conversely, the maximum probability values occurred only around 1300 seconds. This high sensitivity is in line with [1], where the HAI was noted to change rapidly.

### Effect of administered stimulus type on high arousal index

#### Highest arousal stimuli matched self-reported data.

**Fig 5:**
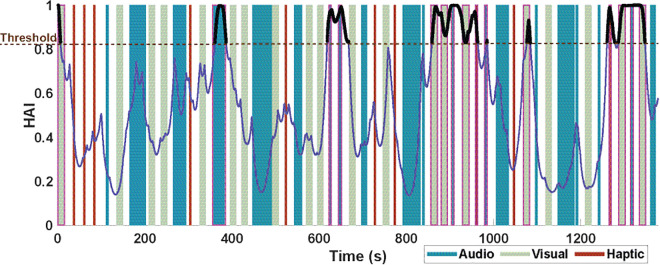
High Arousal Index and stimuli over time for Participant 1. The threshold for high HAI is indicated with a dotted brown line, and the HAI estimates above the threshold are shown with a thick black line. Stimuli that overlap with high HAI are outlined in pink to indicate they are selected for the high HAI set for this participant. The stimuli are color-coded by type.

We identified the stimuli that produced the highest cognitive arousal levels, the high HAI set, by first finding the fifteen periods of stimuli exposure corresponding to the highest HAI levels for each participant and grouping them by type, and then comparing the number of events of each stimulus type in this set against their respective SAM ratings indicated by the participant, as outlined in and inspired by methods of comparison between self-reported and estimated emotional valence in [36]. An example of this process is shown for Participant 1 in Fig 5, where the threshold was first initialized with a value of 1 and then decreased until fifteen stimuli exposure sessions with HAI values above the threshold were identified, as indicated by the dotted brown line. For stimuli with durations under five seconds, the five-second period after stimulus onset was considered to avoid bias against stimuli of short duration. The threshold for high HAI varied between participants and was governed by the participant-specific value needed to select the fifteen stimuli. The thresholds ranged from the 70^*th*^ to 95^*th*^ percentile of HAI. To evaluate the effectiveness of this model at identifying stimuli that caused high arousal, the overlap between the high HAI set and the user’s highest-rated stimuli was computed. The median overall accuracy of identifying a high arousal stimulus using the high HAI set was 69.0% *± *5.4%. The specificity and sensitivity were 72.7% *± *4.3% and 50.0% *± *18.7%, respectively.

#### Auditory stimuli caused greater high arousal index.

**Fig 6:**
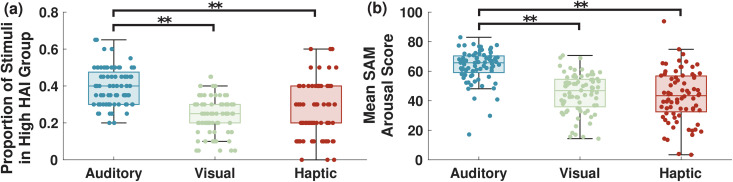
Comparison of computationally identified and self-rated stimuli causing high cognitive arousal. (a) Box plots showing the distributions across participants of the proportion of stimuli identified as high HAI events for each stimulus type. (b) Distribution of normalized mean SAM arousal values across all participants for each stimulus type. Each dot shows the value for an individual participant. Significant differences (p < 0.001) are indicated by **, per the Wilcoxon Signed-Rank test.

The proportion of the selected stimuli in the high HAI set was compared to the overall number of stimuli in each group for each participant, and their distribution is shown in Fig 6(a). We found that auditory stimuli were represented significantly more than the other types in the high HAI group, with p < 0.001 per the Wilcoxon signed-rank test. This result indicated that the auditory stimuli resulted in high arousal significantly more frequently than the other stimulus types. No statistically significant difference was observed between the visual and haptic groups (p > 0.05 per Wilcoxon signed-rank).

These results were compared to the self-reported SAM arousal values from the participants. The mean SAM value for each participant and each stimulus type is shown in Fig 6(b). Notably, the trends in the average SAM score matched the trends in the high HAI sets. Once again, the auditory group had a significantly higher value than the other two stimulus types (p < 0.001 per Wilcoxon signed-rank test), and there was no significant difference between the visual and haptic groups (p > 0.05). These results validated our observations from the high HAI analysis. On average, the auditory stimuli resulted in the highest arousal across participants.

To assess whether participant demographics affected these results, we ran the analysis separately for male and female participants, and found no significant differences in the composition of the high HAI groups, per the Wilcoxon rank-sum test (p > 0.05). Similarly, to assess if age could be a factor, we separated the participants into young adults (age < 30) and older adults (age *≥ *30). Again, there were no significant differences in the high HAI sets of these groups per the Wilcoxon rank-sum test (p > 0.05).

#### High arousal index trends matched self-reported arousal ratings.

**Fig 7:**
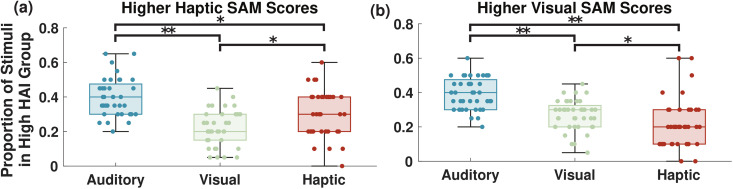
Box plots showing the distributions across participants of the proportion of stimuli identified as high HAI events for each stimulus type. Data from Fig 6 (a) was categorized into two classes: participants with higher average SAM ratings for (a) haptic compared to visual stimuli and (b) visual compared to haptic stimuli. Each dot shows the value for an individual participant. * indicates a significant difference with p < 0.05 and ** indicates a significant difference with p < 0.001, per the Wilcoxon Signed-Rank test.

To further the analysis of the arousal estimates compared to the SAM values, we divided the participants into smaller groups. While nearly all of the participants (93%) had the highest mean SAM rating for the auditory stimuli, the participants showed no significant difference between the visual and haptic stimulus types, with 47% and 53%, respectively, rating the visual and haptic stimuli as causing higher arousal in their SAM scoring. To investigate differences in these groups, the participants were split into two groups: those who rated visual higher and those who rated haptics higher. The proportions of each stimulus type in the high HAI sets are shown for both of these groups in Fig 7. Once again, statistical testing was done via the Wilcoxon signed-rank test. The group of participants with higher average self-reported arousal ratings for the haptic stimuli (Fig 7(a)) had significantly more haptic stimuli exposure periods in their high HAI sets compared to visual stimuli (p < 0.05). Conversely, the group that had higher average self-reported arousal ratings for visual stimuli (Fig 7(b)) had significantly more visual stimuli exposure periods in their high HAI sets (p < 0.05). Thus, this method was able to distinguish between participants with more susceptibility to one stimulus type than another, capturing the self-reported results.

#### Changes in the estimated arousal state matched changes in self-reported arousal ratings.

**Fig 8:**
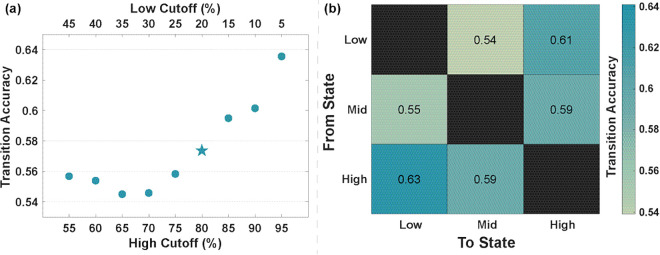
Accuracy of characterizing changes in self-reported stimuli rating based on the change in estimated arousal state for varying cutoffs. (a) Scatter plot of transition accuracy for different cutoffs of high and low arousal. The star at the 80% high arousal cutoff denotes that these are the results shown in more detail in panel. (b) Transition accuracies between each state with the high arousal cutoff at 80% and the low arousal cutoff of 20%. The rows represent the arousal rating transitioned from, and the columns represent the arousal rating transitioned to.

The stimuli were divided into High-, Mid- and Low- arousal according to the mean raw (unnormalized) SAM ratings across participants, as described in the Transitions in Arousal section, with the boundaries between groups determined by high and low cutoff percentiles. The transitions in ANS response to sequential stimuli of different ratings were analyzed to see if the change in arousal estimate matched the change in rating. The results of this analysis are shown in Fig 8. Fig 8(a) shows the overall accuracy results depending on the high and low cutoff percentiles used, varying from the 55^*th*^ to 95^*th*^ percentiles for the high cutoff and 5^*th*^ to 45^*th*^ percentiles for the low cutoff. The accuracies range from 54.5% to 63.6% and are highest for the most extreme cutoff values. Fig 8(b) focuses in on the case with the 80^*th*^ percentile high cutoff and 20^*th*^ low cutoff as an example to consider how the accuracy varies depending on the type of transition. The overall accuracy in transition with these cutoff values across all participants and transition types was 57.6%. Additionally, the accuracy was slightly higher for transitions between the High- and Low-Rated stimuli (62.2%) only compared to transitions that included the Mid-level stimuli (56.7%).

## Discussion

In this work, we presented a method that provides a quantitative description of the participants’ ANS response to stimuli and is objective rather than subjective (SAM ratings). When analyzing the time-normalized norms of the ANS activation, it was found that they were greatest for the period within two seconds of a new stimulus onset, indicating that the ANS reaction to new stimuli primarily occurs within that two-second window. Extending the range further to include the entire period the participant is exposed to the stimulus decreased these values compared to the onset region. This showed that the initial ANS response of the user was not maintained for the duration of the stimulus but rather was concentrated near the stimulus onset.

However, even when considering the entire period of stimulus exposure, there was still a notable increase in the number and magnitude of pulses per second compared to the periods when no stimuli exposure occurred. This indicated that although the response to sustained stimuli may be smaller than a newly applied stimulus, there was still an increase in the ANS activity compared to when no stimuli were applied. Thus, through this method, we were also able to identify the timescales over which the sensory inputs significantly modulated participants’ arousal levels. Since the biggest reaction from the nervous system occurred within the first few seconds after exposure to a new stimulus, a platform intended to invoke high engagement of the ANS may benefit from frequent changes in the visual, auditory, and haptic output.

The analysis of the time-normalized norms of the ANS pulses also revealed the differences in response based on the type of stimuli applied. As expected, for all stimuli types, there was a significant increase in the count, magnitude, and energy of the pulses compared to the case when no stimuli were applied. Moreover, it was found that haptic stimuli corresponded to the greatest ANS responses, followed by auditory and then visual stimuli. In contrast, the stimulus type that produced the greatest increase in estimated arousal state was auditory, as discussed below. The high ANS pulse norms in response to the haptic stimuli may be related to the differences in these stimuli compared to the auditory and haptic stimuli. Namely, the short duration of the stimuli and the method of applying the haptic stimuli (puffs of air on the finger) could influence these results. Future work should consider other types of haptic stimuli to better understand the effect of haptic stimuli on the ANS response.

One potential use for the MPP-based arousal estimation is to determine how the arousal level of a user has changed in response to stimuli. One would expect to see an increase (decrease) in arousal estimate when a participant goes from a stimulus they rated as lower (higher) arousal to one they rated as higher (lower) arousal. Our analysis found that the changes in arousal estimates between stimuli of different ratings followed the trends of the SAM ratings 54.5% to 63.6% of the time, depending on the cutoff values used. This accuracy is higher than 50%, which would be the accuracy expected from randomly guessing whether the arousal increased or decreased at each transition. The accuracy further increased when considering only the larger changes in rating, from Low to High or High to Low (Fig 8). Some of the transitions between Mid and Low or Mid and High could actually be very similar in arousal, differing in arousal rating by only a few points. In these cases, the accuracy may be limited by the quantization and the subjective nature of the ratings, masking small changes in the arousal estimate.

This idea is further supported by considering the trend in accuracy as the cutoff values get more extreme. The accuracy is highest when the high cutoff is at the 95^*th*^ percentile and the low cutoff is at the 5^*th*^ percentile. In this case, all of the transitions between High and Low are very large, so we would expect better accuracy since there is more distinction between High and Low-rated stimuli. However, fewer stimuli fall into these extreme High- and Low-rated categories, so the method may be less useful.

One goal of this work was to develop a method to estimate SAM arousal ratings from the arousal estimates. However, this is a difficult task, as arousal can be impacted by the differences in stimuli modalities, stimuli duration, increased boredom throughout the session, potential delay in ANS response. Additionally, the SAM arousal ratings are subjective and may not be completely accurate to the true arousal state. Thus, it is quite challenging to create an all-encompassing method to predict the SAM ratings from skin conductance, particularly since the SAM ratings are much sparser than the skin conductance signal. This is reflected in the lack of overall correlation between our arousal estimate and the user SAM ratings when considering all stimuli. The average R^2^ value for a linear fit between the arousal estimate and SAM ratings was only 0.03. However, while the overall correlation between the estimated and self-reported arousal was low, the ability of this model to detect stimuli that were rated as high arousal was much better. In future work, a study could be designed to specifically focus on correlating arousal estimate with self-reported arousal, but this study design should incorporate sufficient rest periods between stimuli, consistent stimulus duration and modality, and more frequent self-reports of arousal to capture arousal decay between stimuli, which are all features not present in this dataset.

While it is difficult to find an overall mapping between self-reported arousal from SC signals, the accuracy of this work in predicting which events the users rated as highest arousal validates its use as a detector for high arousal stimuli. Additionally, the high specificity of the method indicates that a stimulus included in the high HAI set is highly likely to be rated highly by the user. However, the lower sensitivity indicates that some highly rated stimuli will be missed in the high HAI set. The non-uniform periods of exposure to each stimulus type may have limited this accuracy, which could be improved in the future by considering uniform exposure duration. The accuracy of this approach could also have been negatively impacted by the time-proximity of the stimuli, which did not allow users to fully return to a baseline level of arousal before continuing, resulting in residual high arousal due to previous stimuli during lower-rated stimuli exposure. Since the stimulus order was randomized for each participant, this effect can be mitigated when considering the entire population.

For each sensory stimulus, we calculated the relative frequency of their representation in the high HAI set. The auditory stimuli were represented most often in this set, in agreement with the SAM arousal scores provided by the participants (Fig 6). It is particularly noteworthy that haptic stimuli had the most ANS response in terms of the time-normalized norms of ANS pulses, but auditory stimuli were most represented in the high HAI set. This is significant, as it indicates that the high HAI set captured the SAM-based insights. Based on this result, we can conclude that auditory stimuli in this experimental setting had a high capability for increasing cognitive arousal.

Furthermore, we also considered the differences in responses between the haptic and visual stimuli. When the whole group was considered, there were no significant differences in the proportion of these two stimulus types in the high HAI set. The SAM scores also reflected this, as participants were split almost evenly between rating visual or haptic as causing higher arousal. However, when considering the participants as two separate groups, those who rated haptics higher and those who rated visual higher, differences in the high HAI sets were revealed. Those who self-reported higher arousal to haptic (visual) stimuli were found to have a higher proportion of haptic (visual) stimuli in their high HAI sets, with statistical significance. This finding shows that our method is not only able to broadly characterize the arousal of a group of participants by stimulus type, but it can also discern differences in the population based on which stimuli they are more responsive to. This indicates that this method could be used not only to design a VR environment for the general public but that it could adapt to the individual user based on their personal responsiveness. This level of customized feedback can enhance the VR environment for all users, with applications such as relaxation, improving cognitive performance, and treatment of anxiety [37–39].

It should be noted that these results comparing the arousal effect of different types of stimuli should be treated with caution. It is difficult to compare and match intensity across different stimulus modalities. While ten each of the auditory and visual stimuli were chosen from the IADS and IAPS databases, respectively, to encompass a range of intensities, the intensity of the remaining stimuli was untested. Additionally, the haptic stimuli were limited to puffs of air on the fingers. The participant responses may differ if the haptic stimuli had been applied to different parts of the body or via a different method. As noted previously, the varying lengths of time that the stimuli were applied further impacts the arousal response. Notably, the short duration of the haptic stimuli could affect the reaction to that stimulus type. The short duration was due to the limitations of the device providing the haptic feedback, which was limited to short puffs of air. However, it should be noted that short-duration stimuli can still result in large ANS responses, as seen in response to electric shocks [29]. To further understand the effect of stimulus types, different types of haptic stimuli and varying durations should be considered in future work. Given the difficulty of fairly comparing cross-modal stimuli, the result that the auditory stimuli corresponded with the highest level of arousal should be considered cautiously. However, the ability of the high HAI set-based method to match user-reported ratings despite these differences in modalities is notable.

One other potential concern for this study is that the effect of boredom may increase throughout the session. However, it should be noted that neither the estimated arousal values nor the SAM ratings showed a correlation with time, indicating that boredom was likely not a major factor. Additionally, since the order of the stimuli was randomized for each participant, the effect of boredom would be mitigated for population-level analyses.

Currently, patients’ self-ratings of symptoms is the gold standard for obtaining patient information, determining care and interventions [40,41]. In [40], a survey was used to evaluate suicidal ideation in 1084 medical hospital staff who were not physicians, while in [41], the Montgomery–Åsberg Depression Rating Scale was used to assess the effectiveness of ketamine treatment in 24 patients with treatment-resistant depression. However, when the symptoms experienced are psychological and hence subjective, objective symptom quantification will (i) reduce biases leading to misdiagnoses, and (ii) help devise appropriate treatment. Such quantification of electrophysiology data, particularly in response to sensory stimuli, will also help evaluate the effectiveness of VR-based sensory stimulation systems in providing symptom relief. Identifying the salient features in measured physiological signals that help track one’s cognitive state is the first step towards this quantification, which we have addressed in this work. In contrast to many works that seek to identify signal characteristics associated with skin conductance data, we identify that the hidden cognitive arousal estimate-based measures are in better congruence with the participant-reported evaluations. In particular, although the analysis of ANS activation events identified the haptic stimuli as causing more average ANS activation than auditory or visual stimuli, the cognitive arousal-based measures identified the auditory stimuli as most arousing, as reported by participants, suggesting their potency as reliable, objective indicators of physiological response to sensory stimuli, enabling future studies involving closed loop interventions. Finally, we note that we have only considered the arousal ratings by participants, while the participants also reported valence and dominance. The study of these other affective states that were also reported in this dataset may reveal additional insights on the nature of emotion perception in the participants.

## Conclusion

In this work, we quantified the skin conductance signal-based ANS activation due to sensory stimuli and the resulting modulations in cognitive arousal. We identified the stimulus that elicits the greatest effect on the (i) ANS response as measured by the sweat secretory events and (ii) cognitive arousal state estimate. We found that the cognitive arousal estimate, and not the inferred ANS activation event-based measures, effectively captured the subjective SAM-based scores on the affective states in response to stimulus perception, offering a novel perspective to capture patients’ perception of affective states and their modulation induced by sensory stimuli. As mentioned previously, this computational analysis can further our understanding of human psychophysiology by providing a plausible replacement for the subjective, state-of-the-art methods such as self-reports. This work can be used in the diagnosis and treatment of mental health conditions as an objective characterization of a patient’s cognitive state alongside patient-reported (subjective) measures. This study can also inspire future investigations of how a VR environment may best modulate one’s cognitive state by providing the quantified impact of sensory stimulation on participants. This can inspire future work involving closed-loop interventions or human-computer interactions (e.g., workplace or VR settings) where the real-time estimates of a user’s cognitive state can be used to modulate the sensory stimuli in a closed-loop manner.

## Supporting information

S1 Table Participant Processing Information. Usability of each participants’ data and parameter settings for the skin conductance deconvolution analysis.
(PDF)

S2 Table Accuracy Results. The High Arousal Index (HAI) threshold used for identifying stimuli that caused high cognitive arousal, and the calculated accuracy, specificity and sensitivity measures quantifying the overlap between the identified high HAI set and the user’s highest-rated stimuli.
(PDF)

S1 Text Mathematical Background. Description of physiological model for skin conductance signal, deconvolution framework and cognitive arousal estimation.
(PDF)

S1 Fig Deconvolution results for participants 1, 2, 3, 4, 7, 9, 10, 11, and 14. Each plot shows the participant’s skin conductance measurements and estimate, tonic skin conductance component, phasic skin conductance component and autonomic nervous system activation events throughout the experiment. The background for skin conductance data is color-coded by administered stimuli as follows: auditory stimuli in dark green, visual stimuli in light green, and haptic stimuli in orange.
(PDF)

S2 Fig Deconvolution results for participants 16, 17, 18, 19, 20, 21, 22, 23, and 24. Each plot shows the participant’s skin conductance measurements and estimate, tonic skin conductance component, phasic skin conductance component and autonomic nervous system activation events throughout the experiment. The background for skin conductance data is color-coded by administered stimuli as follows: auditory stimuli in dark green, visual stimuli in light green, and haptic stimuli in orange.
(PDF)

S3 Fig Deconvolution results for participants 25, 27, 28, 29, 30, 32, 33, 35, and 36. Each plot shows the participant’s skin conductance measurements and estimate, tonic skin conductance component, phasic skin conductance component and autonomic nervous system activation events throughout the experiment. The background for skin conductance data is color-coded by administered stimuli as follows: auditory stimuli in dark green, visual stimuli in light green, and haptic stimuli in orange.
(PDF)

S4 Fig Deconvolution results for participants 37, 38, 39, 40, 41, 42, 43, 44, and 45. Each plot shows the participant’s skin conductance measurements and estimate, tonic skin conductance component, phasic skin conductance component and autonomic nervous system activation events throughout the experiment. The background for skin conductance data is color-coded by administered stimuli as follows: auditory stimuli in dark green, visual stimuli in light green, and haptic stimuli in orange.
(PDF)

S5 Fig Deconvolution results for participants 46, 47, 48, 49, 50, 51, 52, 54, and 56. Each plot shows the participant’s skin conductance measurements and estimate, tonic skin conductance component, phasic skin conductance component and autonomic nervous system activation events throughout the experiment. The background for skin conductance data is color-coded by administered stimuli as follows: auditory stimuli in dark green, visual stimuli in light green, and haptic stimuli in orange.
(PDF)

S6 Fig Deconvolution results for participants 57, 58, 59, 60, 61, 62, 63, 64, 65. Each plot shows the participant’s skin conductance measurements and estimate, tonic skin conductance component, phasic skin conductance component and autonomic nervous system activation events throughout the experiment. The background for skin conductance data is color-coded by administered stimuli as follows: auditory stimuli in dark green, visual stimuli in light green, and haptic stimuli in orange.
(PDF)

S7 Fig Deconvolution results for participants 68, 69, 70, 73, 74, 75, 77, 80, and 84. Each plot shows the participant’s skin conductance measurements and estimate, tonic skin conductance component, phasic skin conductance component and autonomic nervous system activation events throughout the experiment. The background for skin conductance data is color-coded by administered stimuli as follows: auditory stimuli in dark green, visual stimuli in light green, and haptic stimuli in orange.
(PDF)

S8 Fig Deconvolution results for participants 85, 86, 87, 88, 89 91, 92, 93 and 95. Each plot shows the participant’s skin conductance measurements and estimate, tonic skin conductance component, phasic skin conductance component and autonomic nervous system activation events throughout the experiment. The background for skin conductance data is color-coded by administered stimuli as follows: auditory stimuli in dark green, visual stimuli in light green, and haptic stimuli in orange.
(PDF)

S9 Fig Deconvolution results for participants 96, 97, 99, and 100. Each plot shows the participant’s skin conductance measurements and estimate, tonic skin conductance component, phasic skin conductance component and autonomic nervous system activation events throughout the experiment. The background for skin conductance data is color-coded by administered stimuli as follows: auditory stimuli in dark green, visual stimuli in light green, and haptic stimuli in orange.
(PDF)

S10 FigCognitive arousal estimation results 1, 2, 3, 4, 7, 9, 10, 11, and 14.Plots of cognitive arousal estimation results for each participant’s skin conductance data. Each plot shows the skin conductance signal, the underlying autonomic nervous system activation event amplitudes inferred from the deconvolution analysis, cognitive arousal state ($x_{j}$), probability of pulse occurrence ($p_{j}$) and high arousal index (HAI).(PDF)

S11 FigCognitive arousal estimation results 16, 17, 18, 19, 20, 21, 22, 23, and 24.Plots of cognitive arousal estimation results for each participant’s skin conductance data. Each plot shows the skin conductance signal, the underlying autonomic nervous system activation event amplitudes inferred from the deconvolution analysis, cognitive arousal state ($x_{j}$), probability of pulse occurrence ($p_{j}$) and high arousal index (HAI).(PDF)

S12 FigCognitive arousal estimation results 25, 27, 28, 29, 30, 32, 33, 35, and 36.Plots of cognitive arousal estimation results for each participant’s skin conductance data. Each plot shows the skin conductance signal, the underlying autonomic nervous system activation event amplitudes inferred from the deconvolution analysis, cognitive arousal state ($x_{j}$), probability of pulse occurrence ($p_{j}$) and high arousal index (HAI).(PDF)

S13 FigCognitive arousal estimation results 37, 38, 39, 40, 41, 42, 43, 44, and 45.Plots of cognitive arousal estimation results for each participant’s skin conductance data. Each plot shows the skin conductance signal, the underlying autonomic nervous system activation event amplitudes inferred from the deconvolution analysis, cognitive arousal state ($x_{j}$), probability of pulse occurrence ($p_{j}$) and high arousal index (HAI).(PDF)

S14 FigCognitive arousal estimation results 46, 47, 48, 49, 50, 51, 52, 54, and 56.Plots of cognitive arousal estimation results for each participant’s skin conductance data. Each plot shows the skin conductance signal, the underlying autonomic nervous system activation event amplitudes inferred from the deconvolution analysis, cognitive arousal state ($x_{j}$), probability of pulse occurrence ($p_{j}$) and high arousal index (HAI).(PDF)

S15 FigCognitive arousal estimation results 57, 58, 59, 60, 61, 62, 63, 64, and 65.Plots of cognitive arousal estimation results for each participant’s skin conductance data. Each plot shows the skin conductance signal, the underlying autonomic nervous system activation event amplitudes inferred from the deconvolution analysis, cognitive arousal state ($x_{j}$), probability of pulse occurrence ($p_{j}$) and high arousal index (HAI).(PDF)

S16 FigCognitive arousal estimation results 68, 69, 70, 73, 74, 75, 77, 80, and 84.of cognitive arousal estimation results for each participant’s skin conductance data. Each plot shows the skin conductance signal, the underlying autonomic nervous system activation event amplitudes inferred from the deconvolution analysis, cognitive arousal state ($x_{j}$), probability of pulse occurrence ($p_{j}$) and high arousal index (HAI).(PDF)

S17 FigCognitive arousal estimation results 85, 86, 87, 88, 89 91, 92, 93 and 95.Plots of cognitive arousal estimation results for each participant’s skin conductance data. Each plot shows the skin conductance signal, the underlying autonomic nervous system activation event amplitudes inferred from the deconvolution analysis, cognitive arousal state ($x_{j}$), probability of pulse occurrence ($p_{j}$) and high arousal index (HAI).(PDF)

S18 FigCognitive arousal estimation results 96, 97, 99, and 100.Plots of cognitive arousal estimation results for each participant’s skin conductance data. Each plot shows the skin conductance signal, the underlying autonomic nervous system activation event amplitudes inferred from the deconvolution analysis, cognitive arousal state ($x_{j}$), probability of pulse occurrence ($p_{j}$) and high arousal index (HAI).(PDF)
