# Supplementary material for: Cognitive arousal-based measures quantify insights from self-ratings in response to sensory stimuli
Source: PLOS Ment Health. 2025 Nov 12;2(11):e0000463. doi: 10.1371/journal.pmen.0000463 (PMC12798639; doi:10.1371/journal.pmen.0000463)
Supplement: S1 Table — Usability of each participants’ data and parameter settings for the skin conductance deconvolution analysis. (PDF) [file pmen.0000463.s001.pdf]

S1 Table. Participant Processing Information The usability of the 100 participants' data from the original dataset in [1], the skin conductance model parameter initialization settings and the initial values of tonic and phasic skin conductance are indicated in the table S1.1. The skin conductance model parameter initializations were chosen from among the following four parameter sets. The 'Deconvolution Settings' column in the table S1.1 indicates which initial parameter set from the four listed below was used for each participant.

1. $\tau_r = 10s, \tau_p = 100s, \tau_d = 50000s$
2. $\tau_r = 10s, \tau_p = 500s, \tau_d = 30000s$
3. $\tau_r = 10s, \tau_p = 300s, \tau_d = 20000s$
4. $\tau_r = 50s, \tau_p = 100s, \tau_d = 2500s$

Table A. Usability of participant data in the original dataset [1] and deconvolution parameter settings (τ_r, τ_p, τ_d and initial phasic and tonic components) in the skin conductance model for data analysis.

No.	Usable	Deconvolution Settings	Initial Tonic Component (%)	Initial Phasic Component (%)
1	Yes	4	80	20
2	Yes	4	99	0
3	Yes	3	99	0
4	Yes	3	99	0
5	No - Poor Data Quality	-	-	-
6	No - Poor Data Quality	-	-	-
7	Yes	3	85	15
8	No - Poor Data Quality	-	-	-
9	Yes	2	99	0
10	Yes	4	90	10
11	Yes	4	80	20
12	No - Incomplete Data	-	-	-
13	No - Poor Data Quality	-	-	-
14	Yes	4	75	27
15	No - Poor Data Quality	-	-	-
16	Yes	3	99	0
17	Yes	4	80	20
18	Yes	1	95	5
19	Yes	1	90	10
20	Yes*	3	99	0
21	Yes	3	99	0
22	Yes	1	90	10
23	Yes	4	85	15
24	Yes	4	75	25
25	Yes	4	75	25
26	No - Poor Data Quality	-	-	-
27	Yes	3	99	0

No.	Usable	Deconvolution Settings	Initial Tonic Component (%)	Initial Phasic Component (%)
28	Yes	4	90	10
29	Yes	4	80	20
30	Yes	4	80	20
31	No - Poor Data Quality	-	99	0
32	Yes	4	95	5
33	Yes	4	85	15
34	No - Poor Data Quality	-	-	-
35	Yes	1	99	0
36	Yes	4	85	15
37	Yes	3	95	5
38	Yes	3	85	15
39	Yes	1	99	0
40	Yes	3	85	15
41	Yes	1	95	5
42	Yes	2	99	0
43	Yes	4	90	10
44	Yes	4	85	15
45	Yes	3	85	15
46	Yes	1	99	0
47	Yes	3	85	15
48	Yes	1	92	8
49	Yes	3	90	10
50	Yes	4	99	0
51	Yes	4	99	0
52	Yes	2	85	15
53	No - Incomplete Data	-	-	-
54	Yes	4	90	10
55	No - Poor Data Quality	3	83	17
56	Yes	4	95	5
57	Yes	4	90	10
58	Yes	4	99	0
59	Yes	1	99	0
60	Yes	4	90	10
61	Yes	1	99	0
62	Yes	3	80	20
63	Yes	3	85	15
64	Yes	3	85	15
65	Yes	4	90	10
66	No - Incomplete Data	-	-	-
67	No - Poor Data Quality	-	-	-
68	Yes	1	99	0
69	Yes	4	85	15
70	Yes	4	80	20
71	No - Poor Data Quality	-	-	-
72	No - Poor Data Quality	-	-	-
73	Yes	1	99	0
74	Yes	4	90	10
75	Yes	3	95	5

No.	Usable	Deconvolution Settings	Initial Tonic Component (%)	Initial Phasic Component (%)
76	No - Incomplete Data	-	-	-
77	Yes	4	90	10
78	No - Poor Data Quality	-	-	-
79	No - Poor Data Quality	-	-	-
80	Yes	4	95	5
81	No - Incomplete Data	-	-	-
82	No - Incomplete Data	-	-	-
83	No - Incomplete Data	-	-	-
84	Yes	4	75	25
85	Yes	4	90	10
86	Yes	4	90	10
87	Yes	4	90	10
88	Yes	1	95	5
89	Yes	4	80	20
90	No - Incomplete Data	-	-	-
91	Yes	3	80	20
92	Yes	3	80	20
93	Yes	4	90	10
94	No - Poor Data Quality	-	-	-
95	Yes	2	99	0
96	Yes	3	99	0
97	Yes	4	99	0
98	No - Incomplete Data	-	-	-
99	Yes	3	85	15
100	Yes	3	85	15

*Trimmed first and last five seconds to remove artifact in skin conductance measurements.

References

1. Gatti E, Calzolari E, Maggioni E, Obrist M. Emotional ratings and skin conductance response to visual, auditory and haptic stimuli. Scientific data. 2018;5(1):1–12.
