# Supplementary material for: Cognitive arousal-based measures quantify insights from self-ratings in response to sensory stimuli
Source: PLOS Ment Health. 2025 Nov 12;2(11):e0000463. doi: 10.1371/journal.pmen.0000463 (PMC12798639; doi:10.1371/journal.pmen.0000463)
Supplement: S2 Table — The High Arousal Index (HAI) threshold used for identifying stimuli that caused high cognitive arousal, and the calculated accuracy, specificity and sensitivity measures quantifying the overlap between the identified high HAI set and the user’s highest-rated stimuli. (PDF) [file pmen.0000463.s002.pdf]

S2 Table. Accuracy Results. Cognitive arousal, and the calculated accuracy, specificity and sensitivity measures quantifying the overlap between the identified high HAI set and the user's highest-rated stimuli. To evaluate the effectiveness of our computational method in identifying stimuli that caused high arousal, the identified stimuli with the high arousal index (HAI) over a threshold were compared with the user's highest-rated stimuli. The threshold for each participant and the accuracy, specificity and sensitivity of identification of high arousal-causing stimuli are indicated in Table .

No.	High HAI Threshold (%)	Accuracy (%)	Specificity (%)	Sensitivity (%)
1	73	74	75	67
2	88	66	70	33
3	61	64	69	20
4	84	76	78	67
7	85	76	75	83
9	85	72	71	80
10	51	68	77	47
11	79	62	68	17
14	80	74	74	71
16	78	72	74	57
17	74	66	73	53
18	77	70	72	57
19	67	70	72	57
20	66	74	75	67
21	72	70	75	50
22	82	66	73	40
23	88	68	73	44
24	51	68	70	50
25	74	70	76	54
27	68	66	71	44
28	88	70	71	60
29	50	66	70	43
30	89	72	73	60
32	59	66	73	46
33	79	76	76	80
35	94	80	78	100
36	80	70	74	50
37	52	66	71	38
38	58	84	83	90
39	91	70	73	50
40	66	62	73	41
41	69	72	74	57
42	77	64	71	33
43	75	72	73	60
44	85	68	71	40
45	77	64	70	29
46	99	60	67	14
47	74	64	71	33

No.	High HAI Threshold (%)	Accuracy (%)	Specificity (%)	Sensitivity (%)
48	78	64	70	29
49	81	70	72	57
50	74	68	72	43
51	78	72	76	56
52	91	70	73	50
54	84	76	79	64
56	80	72	74	57
57	67	62	70	38
58	85	58	67	13
59	86	60	67	14
60	89	66	70	33
61	79	76	78	67
62	58	68	71	40
63	73	74	73	80
64	68	64	73	38
65	71	76	81	62
68	56	68	73	44
69	92	66	69	40
70	72	72	73	67
73	70	68	74	45
74	87	64	68	44
75	74	84	97	67
77	75	72	72	71
80	86	62	67	38
84	69	70	75	50
85	84	66	71	38
86	74	74	76	67
87	94	62	67	20
88	79	72	73	60
89	81	64	70	40
91	85	64	69	20
92	81	72	73	60
93	79	68	71	40
95	78	70	77	53
96	68	70	73	56
97	98	54	64	0
99	86	68	71	40
100	79	70	71	60
