# Supplementary material for: Cognitive arousal-based measures quantify insights from self-ratings in response to sensory stimuli
Source: PLOS Ment Health. 2025 Nov 12;2(11):e0000463. doi: 10.1371/journal.pmen.0000463 (PMC12798639; doi:10.1371/journal.pmen.0000463)
Supplement: S1 Text — Description of physiological model for skin conductance signal, deconvolution framework and cognitive arousal estimation. (PDF) [file pmen.0000463.s003.pdf]

S1 Text. Mathematical Background Heightened ANS activation results in rapid production of sweat, which causes fast, yet small changes in the skin conductance (SC). The resulting SC alterations can be measured through sensors on the surface of the skin, and this signal has been shown to be a viable, near real-time indicator of cognitive arousal [1]. Many signal-processing methods have been used in previous work to analyze the SC measurements. A heuristic sigmoid-exponential method was used in [2] to characterize the waveform of the SC measurements; however, this type of model cannot recover the activations behind the rises of the waveform. Differential equation methods such as in [3] use bi-exponential functions but only capture the fast-varying phasic component of SC measurements. Some works separate the fast-varying phasic component and the slow-varying tonic component with Finite Impulse Response (FIR)-based filters before processing each [4]. These methods aim to separate tonic and phasic components and process each accordingly; however, this separation can be better achieved using model-based deconvolution methods [5].

Physiologically plausible methods for inferring the ANS activation from SC have been recently proposed [1, 6–12]. This model formulation allows for the separation of the phasic and tonic components of the SC response and places constraints on the model parameters to ensure they are biologically plausible. This technique has been used in prior studies to test the ANS response to different emotions, such as stress and fear [11, 12], and sensory inputs such as music, perfume, and coffee [13–16].

The topic of emotional response to different stimulus types has been studied previously [17–20]. These studies often utilize the Self-Assessment Manikin (SAM) framework as a measurement of the participant's emotions. This approach entails the indication of one's self-perceived affective states, such as cognitive arousal, valence, or dominance, by choosing their intensity from pictorial depictions. However, these ratings can be difficult to interpret due to their subjective nature and potential bias from the participants. Furthermore, these studies tend to focus on directly measurable physiological responses, such as skin conductance and heart rate, and do not use the state-space approach to quantify the underlying ANS response. This results in a gap in the research as the in-depth information about ANS activity can strengthen our understanding of how the body reacts to different stimuli. Additionally, many of these previous studies focus exclusively on eliciting a single negative emotion, such as fear or disgust [17, 18, 21], although others cover a range of emotions [19], without considerations of their resolution into the basis cognitive states such as the cognitive arousal, valence, and dominance.

S2.1 Model and Deconvolution In this work, we used the poral valve model, proposed in [22], to model the skin conductance response (Fig. 1 in main manuscript). In this scheme, the input of the system ($u(t)$) is an ANS activation event, which causes sweat generation. The system output is the skin conductance response ($y(t)$), which is measured. There are three states in the model, corresponding to the sweat in the sweat ducts ($z_1(t)$), the sweat in the ducts but close enough to the surface to be electrically conductive ($z_2(t)$), and the sweat on the surface of the skin ($z_3(t)$). The relationship between the states and the input and output of the system is given by:

$$A = \begin{bmatrix} \frac{-1}{\tau_r} & 0 & 0 \\ \frac{1}{\tau_r} & \frac{-1}{\tau_p} & 0 \\ \frac{1}{\tau_r} & 0 & \frac{-1}{\tau_d} \end{bmatrix}$$

$$B = \begin{bmatrix} 1 \\ 0 \\ 0 \end{bmatrix}$$

$$C = \begin{bmatrix} 0 & \chi_p & \chi_d \end{bmatrix}$$

$$\dot{\vec{z}}(t) = A\vec{z}(t) + Bu(t)$$
$$y(t) = C\vec{z}(t) + \nu(t)$$

where $\vec{z}(t) = \begin{bmatrix} z_1(t) & z_2(t) & z_3(t) \end{bmatrix}^T$, $\nu(t)$ is an error term, and $\tau_r, \tau_p, \tau_d, \chi_p$ and χ_d are subject-specific parameters, representing slow and fast rise times, decay time and the fractions of phasic and tonic skin conductance components, to be solved for. Note that $\chi_p + \chi_d = 1$. To solve for these parameters, and reconstruct the input to the system, we followed the approach proposed in [1]. We set constant values of $\chi_p = 0.09$ and $\chi_d = 0.91$ for all subjects to reduce the complexity of the computation. To solve for the remaining parameters and to reconstruct the input of the system, we used an expectation-maximization approach.

Since the individual skin conductance parameters can vary widely between individuals, four sets of priors were devised and tested on all subjects. The results for each subject were examined, and the set used for each subject was chosen to (1) ensure that the phasic component remains positive throughout the experiment and (2) ensure there is no overall trend in the phasic component (any slow varying trend should be accounted for in the tonic component). Additionally, the initial proportions of the tonic and phasic components were selected individually for each subject, based on the same criteria. The list of parameter sets can be found in the supplementary material, along with which set of parameters and initial conditions were used for each subject. For all subjects, the variances for these parameters were set to: $\sigma_r^2 = 0.6, \sigma_p^2 = 50, \sigma_d^2 = 22500$. The sensor-specific measurement noise variance, σ_v^2, was set to 10^{-6}. More details on this approach can be found in [1].

S2.2 Cognitive Arousal Estimation Using Marked Point Process-based Filtering The results of the deconvolution step were used as input to estimate the cognitive arousal state of the subject, using a marked point process (MPP)-based filtering approach, where the recovered ANS activation events were treated as a marked point process [23].

In the absence of a stimulus, the cognitive arousal state (x_j) is assumed to vary via a random walk model. Thus, it can be modelled as $x_{j+1} = x_j + \epsilon_j$, where $\epsilon_j \sim \mathcal{N}(0, \sigma_\epsilon^2)$ is the process noise. σ_ϵ is a parameter that must be estimated. The probability (p_j) of an ANS pulse occurring at step j varies with the cognitive arousal. To model this relationship, we use $p_j = \frac{1}{exp(-(\beta + x_j))}$, where β is a constant. In this work, we set β based on the probability of an ANS pulse while the subject is in a neutral state. That is $\beta = log(\frac{p_0}{1-p_0})$, where p_0 is given by the number of ANS pulses divided by the number of time steps in a low cognitive arousal segment of the data. We calculated p_0 using the one-minute data segment in the experiment where the subject reported the lowest arousal.

The amplitude of each ANS pulse should also be accounted for. In this work, as in previous works, we assumed a linear relationship between the magnitude of the pulse r_j and the cognitive arousal state. Thus, these values are related by $r_j = \gamma_0 + \gamma_1 x_j + \nu_j$, where $\nu_j \sim \mathcal{N}(0, \sigma_v^2)$ is the sensor noise and σ_v, γ_0 and γ_1 are subject-specific parameters that need to be estimated. Thus, the joint density function of the observed ANS activation events can be written as:

$$p(n_j \cap r_j | x_j) = \begin{cases} 1 - p_j & \text{if} \quad n_j = 0 \\ p_j \frac{1}{\sqrt{2\pi\sigma_v^2 exp(-\frac{(r_j - \gamma_0 - \gamma_1 x_j)^2}{2\sigma_v^2})}} & \text{if} \quad n_j = 1 \end{cases}$$

To solve for the parameters σ_ϵ^2, σ_v^2, γ_0, and γ_1, as well as the cognitive arousal state, z_j, we use an expectation-maximization algorithm. The initial values used in this approach were: $\sigma_\epsilon^2 = 0.06$, $\sigma_v^2 = 0.02$, $\gamma_0 = 0.03$, and $\gamma_1 = 0.01$. The cognitive arousal estimate at the first step was initialized to zero.

In addition to producing an estimate of cognitive arousal and the probability of occurrence of an ANS pulse over time, the High Arousal Index (HAI) was also computed for each subject throughout the experiment. The HAI is equal to the probability that the cognitive arousal state (at a given time) is greater than the median cognitive arousal state for that user ($HAI_j = p(x_j > median(\vec{x})$, where $\vec{x}$ is the vector of cognitive arousal estimates at each time step). This property was originally inspired by the ideal observer certainty level described in [24]. It is used to determine which regions can be marked as high cognitive arousal with high confidence. Further details on this approach are provided in [23].

References

1. Amin R, Faghih RT. Physiological characterization of electrodermal activity enables scalable near real-time autonomic nervous system activation inference. PLoS computational biology. 2022;18(7):e1010275.

2. Lim CL, Rennie C, Barry RJ, Bahramali H, Lazzaro I, Manor B, et al. Decomposing skin conductance into tonic and phasic components. Int J Psychophysiol. 1997;25(2):97–109.

3. Alexander DM, Trengove C, Johnston P, Cooper T, August JP, Gordon E. Separating individual skin conductance responses in a short interstimulus-interval paradigm. J Neurosci Methods. 2005;146(1):116–123.

4. Subramanian S, Barbieri R, Brown EN. Point process temporal structure characterizes electrodermal activity. Proceedings of the National Academy of Sciences. 2020;117(42):26422–26428. doi:10.1073/pnas.2004403117.

5. Benedek M, Kaernbach C. Decomposition of skin conductance data by means of nonnegative deconvolution. Psychophysiology. 2010;doi:10.1111/j.1469-8986.2009.00972.x.

6. Amin MR, Faghih RT. Identification of sympathetic nervous system activation from skin conductance: A sparse decomposition approach with physiological priors. IEEE Transactions on Biomedical Engineering. 2020;68(5):1726–1736.

7. Amin MR, Faghih RT. Tonic and phasic decomposition of skin conductance data: A generalized-cross-validation-based block coordinate descent approach. In: 2019 41st Annual International Conference of the IEEE Engineering in Medicine and Biology Society (EMBC). IEEE; 2019. p. 745–749.

8. Amin MR, Faghih RT. Sparse deconvolution of electrodermal activity via continuous-time system identification. IEEE Transactions on Biomedical Engineering. 2019;66(9):2585–2595.

9. Amin MR, Faghih RT. Robust inference of autonomic nervous system activation using skin conductance measurements: A multi-channel sparse system identification approach. IEEE Access. 2019;7:173419–173437.

10. Amin MR, Faghih RT. Inferring autonomic nervous system stimulation from hand and foot skin conductance measurements. In: 2018 52nd Asilomar Conference on Signals, Systems, and Computers. IEEE; 2018. p. 655–660.

11. Wickramasuriya DS, Qi C, Faghih RT. A state-space approach for detecting stress from electrodermal activity. In: 2018 40th Annual International Conference of the IEEE Engineering in Medicine and Biology Society (EMBC). IEEE; 2018. p. 3562–3567.

12. Wickramasuriya DS, Faghih RT. A Bayesian filtering approach for tracking arousal from binary and continuous skin conductance features. IEEE Transactions on Biomedical Engineering. 2019;67(6):1749–1760.

13. Fekri Azgomi H, F Branco LR, Amin MR, Khazaei S, Faghih RT. Regulation of brain cognitive states through auditory, gustatory, and olfactory stimulation with wearable monitoring. Scientific reports. 2023;13(1):12399.

14. Amin MR, Tahir M, Faghih RT. A State-space Investigation of Impact of Music on Cognitive Performance during a Working Memory Experiment. In: 2021 43rd Annual International Conference of the IEEE Engineering in Medicine & Biology Society (EMBC). IEEE; 2021. p. 757–762.

15. Khazaei S, Amin MR, Faghih RT. Decoding a Neurofeedback-Modulated Performance State in Presence of a Time-Varying Process Noise Variance. In: 2022 56th Asilomar Conference on Signals, Systems, and Computers. IEEE; 2022. p. 990–996.

16. Khazaei S, Amin MR, Faghih RT. Decoding a Neurofeedback-Modulated Cognitive Arousal State to Investigate Performance Regulation by the Yerkes-Dodson Law. In: 2021 43rd Annual International Conference of the IEEE Engineering in Medicine & Biology Society (EMBC). IEEE; 2021. p. 6551–6557.

17. Croy I, Laqua K, Süß F, Joraschky P, Ziemssen T, Hummel T. The sensory channel of presentation alters subjective ratings and autonomic responses toward disgusting stimuli—Blood pressure, heart rate and skin conductance in response to visual, auditory, haptic and olfactory presented disgusting stimuli. Frontiers in Human Neuroscience. 2013;7:510.

18. Sjouwerman R, Lonsdorf T. Latency of skin conductance responses across stimulus modalities. Psychophysiology. 2019;56(4):e13307.

19. Li D, Yang Z, Hou F, Kang Q, Liu S, Song Y, et al. EEG-based emotion recognition with haptic vibration by a feature fusion method. IEEE Transactions on Instrumentation and Measurement. 2022;71:1–11.

20. Grewe O, Katzur B, Kopiez R, Altenmüller E. Chills in different sensory domains: Frisson elicited by acoustical, visual, tactile and gustatory stimuli. Psychology of Music. 2011;39(2):220–239.

21. Storm H. Changes in skin conductance as a tool to monitor nociceptive stimulation and pain. Current Opinion in Anaesthesiology. 2008;21(6):796–804. doi:10.1097/aco.0b013e3283183fe4.

22. Edelberg R. Electrodermal mechanisms: A critique of the two-effector hypothesis and a proposed replacement. Progress in electrodermal research. 1993; p. 7–29.

23. Wickramasuriya DS, Faghih RT. A marked point process filtering approach for tracking sympathetic arousal from skin conductance. IEEE Access. 2020;8:68499–68513.

24. Smith AC, Frank LM, Wirth S, Yanike M, Hu D, Kubota Y, et al. Dynamic analysis of learning in behavioral experiments. Journal of Neuroscience. 2004;24(2):447–461.
