# Supplementary material for: Cognitive arousal-based measures quantify insights from self-ratings in response to sensory stimuli
Source: PLOS Ment Health. 2025 Nov 12;2(11):e0000463. doi: 10.1371/journal.pmen.0000463 (PMC12798639; doi:10.1371/journal.pmen.0000463)

S8 Figure. Deconvolution results for participants 85, 86, 87, 88, 89 91, 92, 93 and 95. Each plot shows the participant's skin conductance measurements and estimate, tonic skin conductance component, phasic skin conductance component and autonomic nervous system activation events throughout the experiment. The background for skin conductance data is color-coded by administered stimuli as follows: auditory stimuli in dark green, visual stimuli in light green, and haptic stimuli in orange.

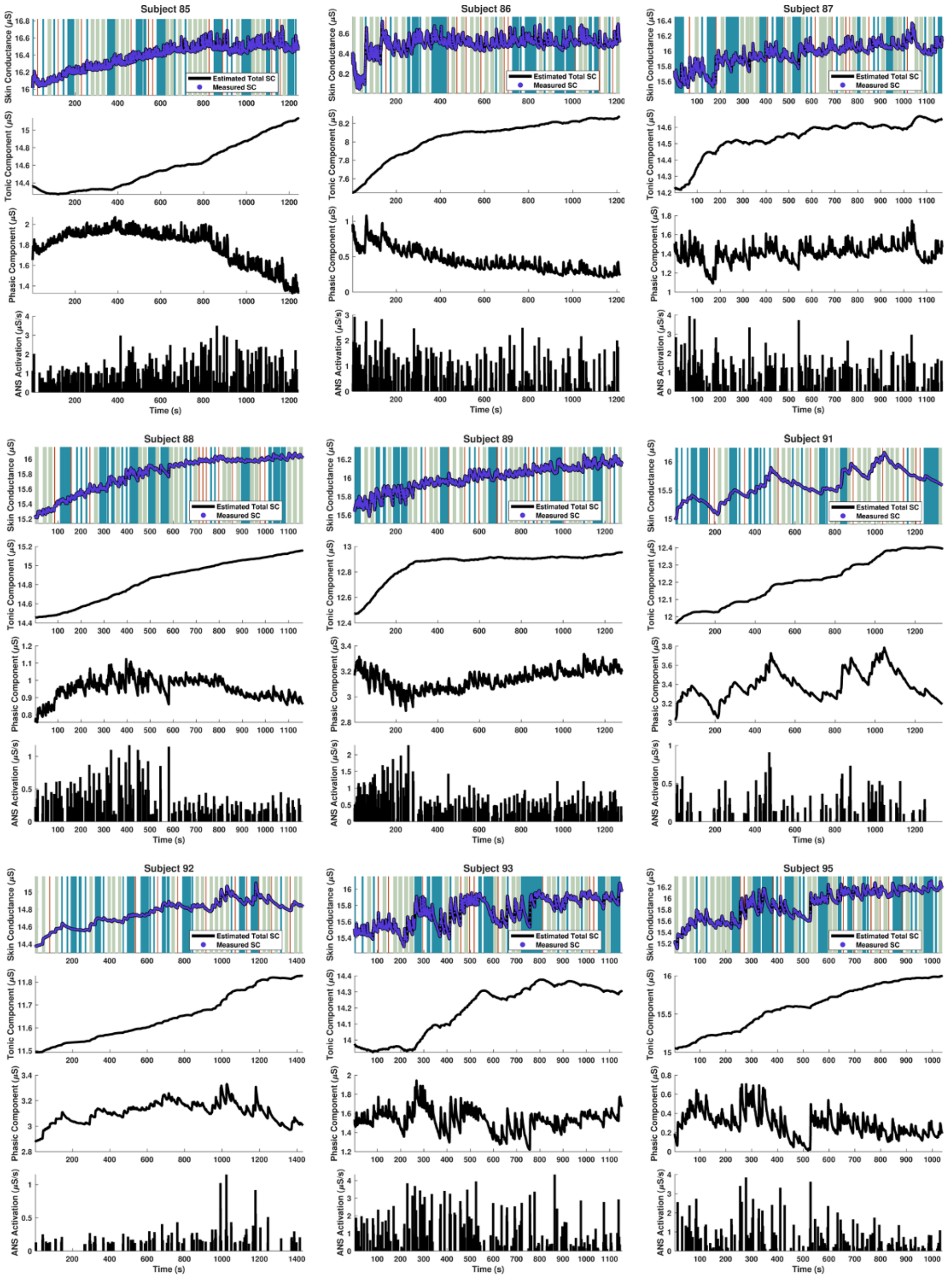

Supplement: S8 Fig — Each plot shows the participant’s skin conductance measurements and estimate, tonic skin conductance component, phasic skin conductance component and autonomic nervous system activation events throughout the experiment. The background for skin conductance data is color-coded by administered stimuli as follows: auditory stimuli in dark green, visual stimuli in light green, and haptic stimuli in orange. (PDF) [file pmen.0000463.s011.pdf]
