# Supplementary material for: Cognitive arousal-based measures quantify insights from self-ratings in response to sensory stimuli
Source: PLOS Ment Health. 2025 Nov 12;2(11):e0000463. doi: 10.1371/journal.pmen.0000463 (PMC12798639; doi:10.1371/journal.pmen.0000463)

S10 Figure. Cognitive arousal estimation results 1, 2, 3, 4, 7, 9, 10, 11, and 14. Plots of cognitive arousal estimation results for each participant's skin conductance data. Each plot shows the skin conductance signal, the underlying autonomic nervous system activation event amplitudes inferred from the deconvolution analysis, cognitive arousal state (x_j), probability of pulse occurrence (p_j) and high arousal index (HAI).

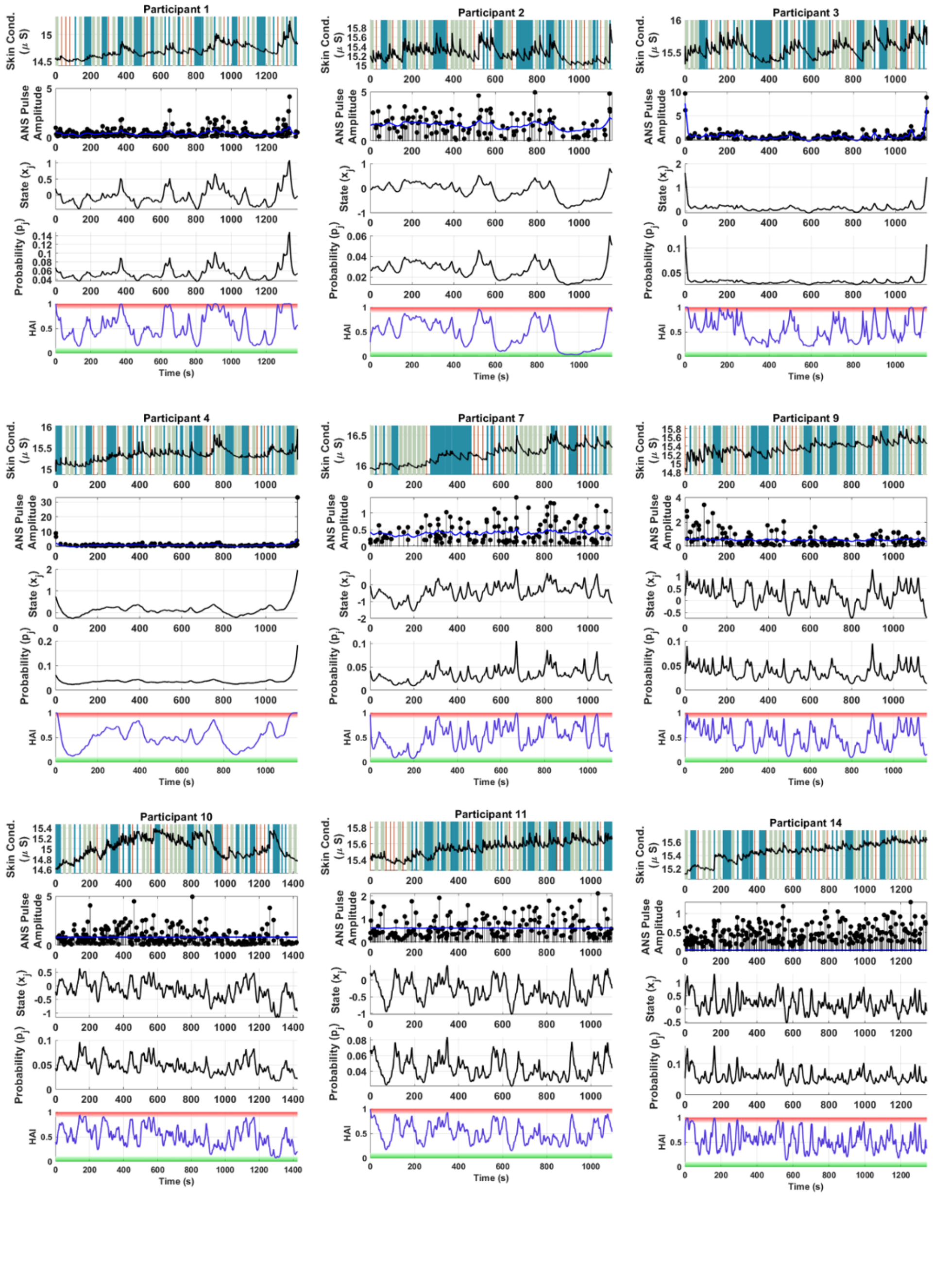

Supplement: S10 Fig — Plots of cognitive arousal estimation results for each participant’s skin conductance data. Each plot shows the skin conductance signal, the underlying autonomic nervous system activation event amplitudes inferred from the deconvolution analysis, cognitive arousal state (xj), probability of pulse occurrence (pj) and high arousal index (HAI). (PDF) [file pmen.0000463.s013.pdf]
